# Development and Validation of a Liquid-Liquid Phase Separation-Related Gene Signature as Prognostic Biomarker for Low-Grade Gliomas

**DOI:** 10.1155/2022/1487165

**Published:** 2022-09-23

**Authors:** Lidong Ning, Guanyan Zhao, Changji Xie, Huan Lan, Jiefei Chen, Hu Tan, ChengCong Wei, Zhiyu Zhou

**Affiliations:** Department of Neurosurgery, National Hospital of Guangxi Zhuang Autonomous Region, Nanning, 530001 Guangxi, China

## Abstract

**Aim:**

To explore whether the liquid-liquid phase separation- (LLPS-) related genes were potential prognostic markers that could contribute to the further classification of low-grade gliomas (LGGs).

**Methods:**

The LLPS-related genes were subjected to functional enrichment analysis. The univariable, least absolute shrinkage and selection operator, and multivariable stepwise Cox regression analyses were performed to develop an LLPS-related gene signature (GS) in the discovery data set. The biological characteristics of the high-risk LGG were explored using gene set enrichment analysis. Two independent external data sets were used to validate the LLPS-related GS.

**Results:**

LLPS-related genes are involved in multiple important cancer-related biological processes and pathways in LGG. Nine LLPS-related genes were identified to construct the LLPS-related GS, which was significantly associated with the prognosis of LGG patients. The LLPS-related GS could successfully divide patients with LGG into high- and low-risk groups, and the high-risk group showed a poorer prognosis than the low-risk group. Furthermore, the LLPS-related GS was independent of IDH and 1p19q status. Several cancer-related pathways may be more active in high-risk LGGs, such as IL6 JAK STAT3 signaling pathway. The LLPS-related GS was successfully validated with two independent external data sets.

**Conclusion:**

We developed and validated a novel LLPS-related GS for risk stratification of LGG. Our findings may provide more precise management for LGGs and a useful reference for LLPS mechanism to link LGG studies.

## 1. Introduction

Low-grade gliomas (LGGs) account for 10–20% of all primary brain tumors [1]. Following the identification of key molecular alterations (e.g., IDH mutation and 1p/19q codeletion) [2, 3] of LGG, the World Health Organization (WHO) updated the central nervous system (CNS) tumor classification in 2016 [4], and the understanding of LGG behavior has rapidly evolved. Although the conventional WHO grade of I–IV still remains, the term of LGG is now often used to refer to both grade II and III gliomas. Nonetheless, LGG remains a highly heterogeneous disease [5]. Hence, there is much interest in finding other molecular signatures for further classification for identifying the ideal management of LGG.

In the conventional WHO grading system, the morphology of the nucleoli of tumor cells is one of the critical bases. Nucleoli, like other membraneless condensates inside eukaryotic cells, are mainly formed through a liquid-liquid phase separation (LLPS) mechanism [6]. The LLPS represents the dynamic concentration of biomolecules from a homogeneous environment into a relatively dense phase to form a sparse phase and a dense phase [7]. The proteins involved in LLPS aggregates usually have intrinsically disordered regions (IDRs). These IDRs may mediate weak affinity and nonspecific interactions of multiple targets to trigger LLPS [8–10]. Various critical biological processes, including chromatin organization, transcription, autophagy, DNA damage response, and tumorigenesis, have been reported to use LLPS to generate the corresponding membraneless condensates and play their specific functions [11–13]. It was shown that the aberration of liquid-liquid phase separation involves in many human diseases, including neurodegenerative diseases [14] and cancer [15]. Previous studies suggested that LLPS-related gene signature can be used as a prognostic marker for hepatocellular carcinoma [16] and ovarian cancer [17]. Thus, we hypothesized that LLPS-related genes may be potential prognostic markers that could contribute to the further classification of LGG. In the present study, to confirm our hypothesis, we developed an LLPS-related gene signature independent of known features to identify high-risk LGG.

## 2. Materials and Methods

### 2.1. Data Processing

**Figure 1:**
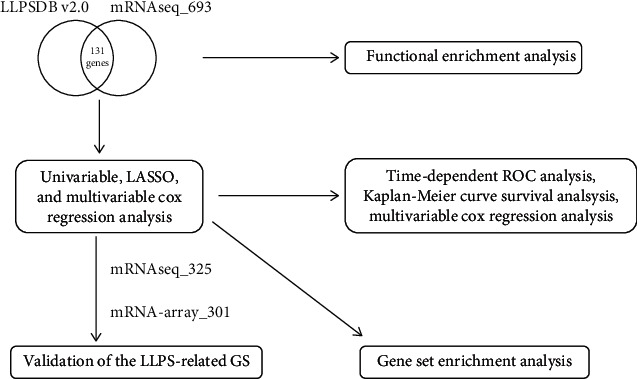
The workflow of the present study.

The gene expression profiles and clinical information of LGG were downloaded from the Chinese Glioma Genome Atlas (http://www.cgga.org.cn/) [18]. This study included three data sets, namely, mRNAseq_693, mRNAseq_325, and mRNA-array_301. The inclusion criteria were as follows: (1) the sample included both gene expression profiles and prognosis information, and (2) the sample was primary LGG (WHO grade II and III glioma). The processed gene expression profiles based on RNA-sequencing technology were normalized used log2 (expression value + 0.01). The LLPS-related genes were obtained from LLPSDB v2.0 [19], and the only the unambiguous genes were included in our present study. As all data of the present are publicly available, ethical approval from the ethics committee of National Hospital of Guangxi Zhuang Autonomous Region was not necessary for the present study. The workflow of the present study is shown Figure 1.

### 2.2. Functional Enrichment Analysis

The 131 genes both in the unambiguous system of LLPSDB v2.0 and mRNAseq_693 data set were identified and performed to functional enrichment analysis. This would help us understand the potential biological functions of LLPS-related genes. The functional enrichment analysis was performed using the clusterProfiler [20] package.

### 2.3. Development of an LLPS-Related Gene Signature for Risk Stratification

Firstly, the univariable Cox regression analysis was performed for screening the prognosis-related genes. Secondly, least absolute shrinkage and selection operator (LASSO) Cox regression was performed for variable selection and shrinkage using glmnet [21] package. The LASSO Cox regression can select the features with a strong predictive value and low correlation between each other to prevent overfitting. In the LASSO analysis, the relevant parameters were set to “family = ^‘^cox′,” “maxit = 1000,” and “nfolds = 10.” Thirdly, the multivariable stepwise Cox regression analysis was applied to develop the LLPS-related gene signature (GS). The LLPS-related GS score for each individual was calculated using the following formula: score = expresion_gene1_∗*β*_gene1_ + expresion_gene2_∗*β*_gene2_ + expresion_gene3_∗*β*_gene3_ + ⋯+expresion_*n*_∗*β*_*n*_.

The prognostic value of the LLPS-related GS score was evaluated using the univariable Cox regression and time-dependent ROC (tROC) curve analysis. The patients with LGG were divided into high-risk and low-risk groups according to the best cutoff, which was identified using the survminer package (https://CRAN.R-project.org/package=survminer). The prognostic value of the novel risk stratification system and other known prognostic features were included in multivariable Cox regression analysis to confirm whether it is an independent prognostic factor.

### 2.4. Gene Set Enrichment Analysis (GSEA)

The GSEA [22, 23] was performed to preliminarily reveal the biological mechanism underlying high-risk LGG. Hallmark gene sets [24] and Kyoto Encyclopedia of Genes and Genomes (KEGG) [25] canonical pathway gene sets were used as reference gene sets. Gene sets with false discovery rate < 0.2 were considered significant enrichment. The GSEA was performed using the GSEA Java software.

### 2.5. Validation of the LLPS-Related GS

The patients of LGG from data sets of mRNAseq_325 and mRNA-array_301 were used as the test sets to validate the LLPS-related GS. Each patient was assigned an LLPS-related GS score according to the above formula. Then, the patients were divided into low- or high-risk groups based on to the best cutoff.

### 2.6. Statistical Analysis

All these analyses were performed using R (version 4.0.2) software (https://www.r-project.org/). The overall survival (OS) between the low- and high-risk groups were compared using the Kaplan-Meier survival curve with log-rank method. We considered *P* values < 0.05 to be statistically significant, unless otherwise stated.

## 3. Results

### 3.1. LLPS-Related Genes Are Involved in Multiple Important Biological Processes and Pathways

**Figure 2:**
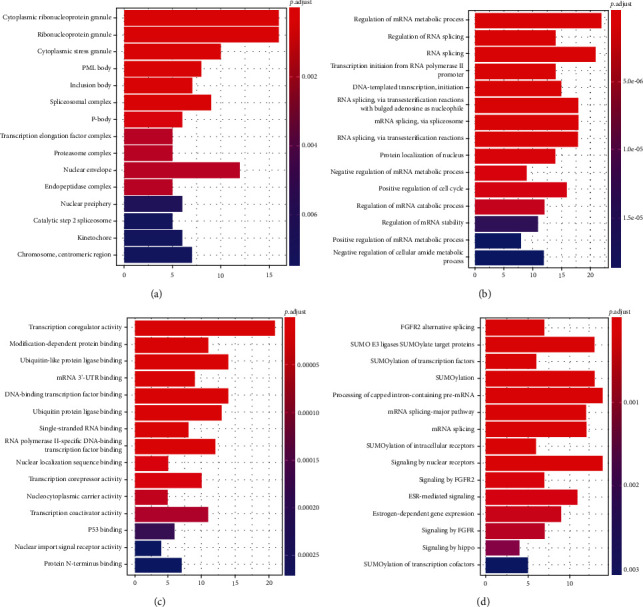
The results of the enrichment analysis of the liquid-liquid phase separation-related genes. (a) Cellular component; (b) biological process; (c) molecular function; (d) reactome pathway.

The results of functional enrichment analysis indicated that the 131 LLPS-related genes are involved in multiple important biological processes and pathways. In the cellular component perspective (Figure 2(a)), the LLPS-related genes were significantly involved in cytoplasmic ribonucleoprotein granule, ribonucleoprotein granule, and cytoplasmic stress granule. In the biological process perspective (Figure 2(b)), the LLPS-related genes were significantly involved in RNA metabolic- and cell cycle-related processes. Molecular functions of the LLPS-related genes include but are not limited to transcription coregulator activity, modification-dependent protein binding, and ubiquitin-like protein ligase binding (Figure 2(c)). The LLPS-related genes involve in multiple cancer-related pathways (Figure 2(d)), such as FGFR2 alternative splicing and mRNA splicing.

### 3.2. LLPS-Related GS as a Novel and Independent Risk Stratification System for LGG

**Figure 3:**
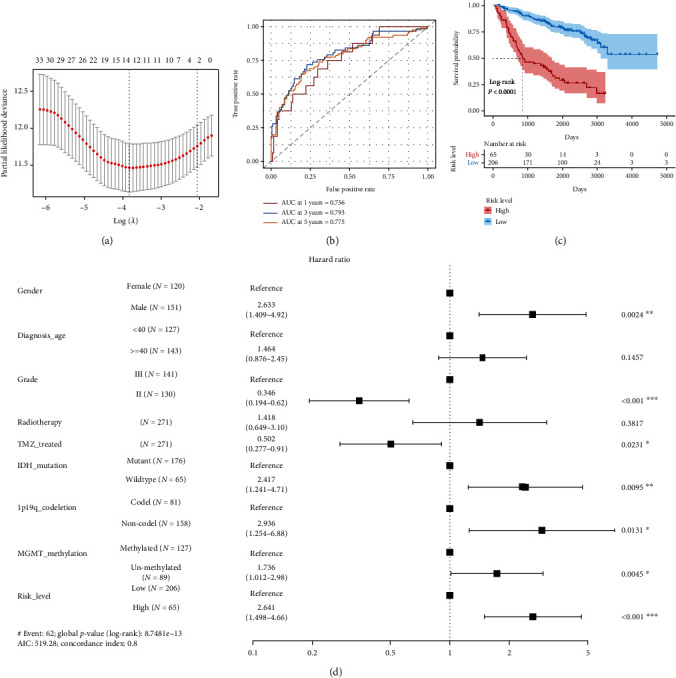
The development of the liquid-liquid phase separation- (LLPS-) related gene signature (GS) in the mRNAseq_693 data sets. (a) Thirteen genes were identified using the least absolute shrinkage and selection operator Cox regression analysis. (b) The time-dependent receiver operating characteristic curve (ROC) analysis. (c) The patients with high LLPS-related GS score had significantly shorter overall survival than those with low LLPS-related GS score. (d) The LLPS-related GS-based risk stratification is a prognostic factor independent of routine clinicopathological characteristics.

**Figure 4:**
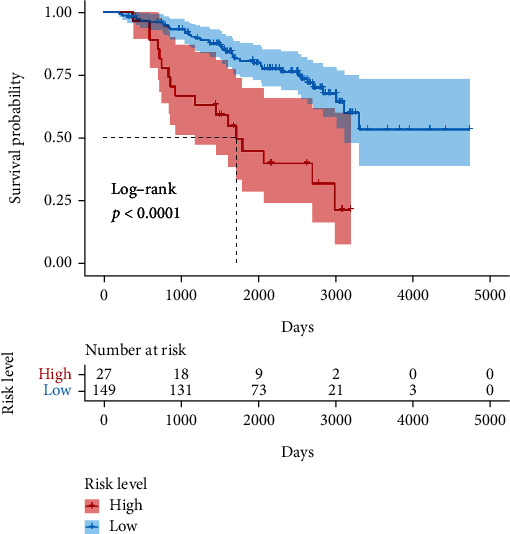
The liquid-liquid phase separation-related gene signature could further risk stratifying IDH-mutant low-grade gliomas.

A total of 271 patients with LGG from mRNAseq_693 data set were included in the development of the LLPS-related GS according to our inclusion criteria. In the univariable Cox regression analysis, thirty-seven LLPS-related genes were considered prognosis-related genes (Table 1). Thirteen LLPS-related genes were identified as nonzero features in the LASSO Cox regression (Table 1 and Figure 3(a)). Finally, nine LLPS-related genes (ABL1, AR, CDK1, DAXX, ELN, KMT2D, POU5F1, SH3KBP1, and SYN1) were identified and used to construct the LLPS-related GS through the multivariable stepwise Cox regression analysis (Table 1). The LLPS-related GS was significantly associated with prognosis (hazard ratio (HR) = 2.718, 95% confidence interval (CI) for HR = 2.185 − 3.382, *P* < 0.001). The tROC curve analysis indicated that the LLPS-related GS may possess a high prognostic value with an area under curve (AUC) of 0.756, 0.793, and 0.775 for 1, 3, and 5 years, respectively (Figure 3(b)). The high-risk patients had significantly shorter OS than the low-risk patients (Figure 3(c)). Furthermore, compared to some other known prognostic factors, the novel risk stratification based on the LLPS-related GS remained a significant prognostic factor (Figure 3(d)). Moreover, the LLPS-related GS could identify high-risk IDH-mutant LGGs (Figure 4). This suggested that our LLPS-related GS could further risk stratifying IDH-mutant LGGs.

### 3.3. High-Risk LGG-Specific Gene Sets

**Figure 5:**
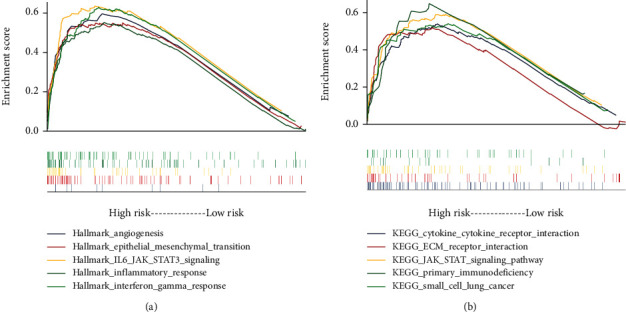
The results of gene set enrichment analysis. (a) The top five (ranked by false discovery rate) hallmark and (b) Kyoto Encyclopedia of Genes and Genomes gene sets were significantly enriched in the high-risk samples.

GSEA showed that the hallmark gene set of “angiogenesis,” “epithelial mesenchymal transition,” “IL6 JAK STAT3 signaling,” “inflammatory response,” and “interferon gamma response” is significantly enriched in the high-risk LGGs (Figure 5(a)). Several cancer-related pathways may be more active in high-risk LGGs (Figure 5(b)), such as IL6 JAK STAT3 signaling pathway and ECM receptor interaction.

### 3.4. The LLPS-Related GS Was Confirmed in Two Independent External Data Sets

**Figure 6:**
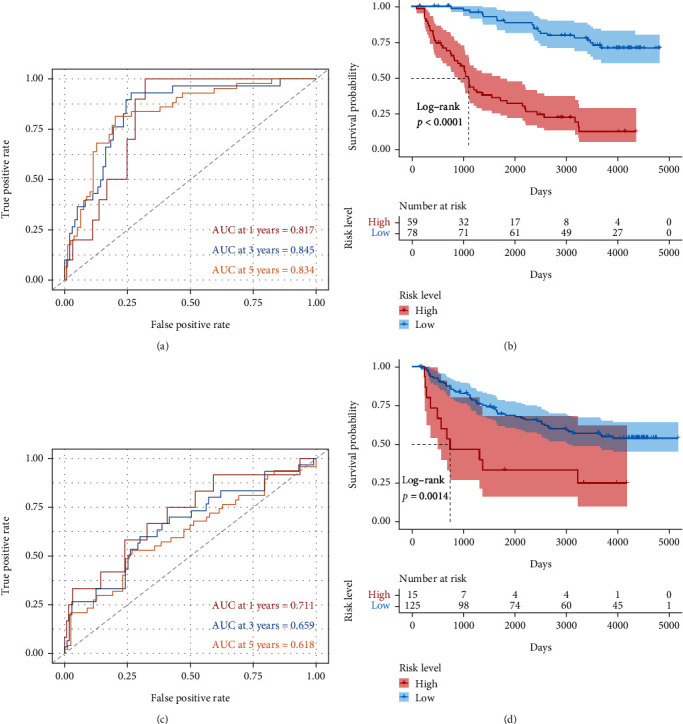
Validation of the liquid-liquid phase separation- (LLPS-) related gene signature (GS). The time-dependent receiver operating characteristic curve (ROC) analysis in (a) mRNAseq_325 and (c) mRNA-array_301. The patients with high LLPS-related GS score had significantly shorter overall survival than those with low LLPS-related GS score both in (b) mRNAseq_325 and (d) mRNA-array_301.

In the data set of mRNAseq_325, 137 patients with LGG were included and validated the LLPS-related GS with HR = 2.390, 95% CI for HR = 1.841 − 3.103, and *P* < 0.001. The tROC curve analysis obtained an AUC of 0.817, 0.845, and 0.834 for 1, 3, and 5 years, respectively (Figure 6(a)). The high-risk patients had significantly shorter OS than the low-risk patients (Figure 6(b)). In the data set of mRNA-array_301, 140 patients with LGG were included and validated the LLPS-related GS with HR = 1.868, 95% CI for HR = 1.120 − 3.114, and *P* < 0.017. The tROC curve analysis obtained an AUC of 0.711, 0.659, and 0.618 for 1, 3, and 5 years, respectively (Figure 6(c)). The high-risk patients had significantly shorter OS than the low-risk patients (Figure 6(d)).

## 4. Discussion

Prediction of prognosis in gliomas was considered much more challenging than with other malignancies. Signs or biomarkers of progression in other malignancies, such as serum CEA and CA199 in colorectal cancer [26], are not present in gliomas. Several clinicopathological features have been considered demonstrating a “higher risk” for progression and poorer prognosis in various studies, including age ≥ 40 years, tumor size > 6 cm, neurological deficits prior to surgery, and tumor that crosses the midline [27, 28]. In recent years, molecular features have been found more reliable than these routine prognostic features. For instance, the isocitrate dehydrogenase (IDH) mutation has been recognized to be correlated to a better prognosis in LGG [29]. The 1p19q codeletion predicts a longer progression-free survival and overall survival [30, 31]. In our present study, we developed and validated a novel molecular signature for predicting the prognosis of LGG. The LLPS-related GS was also independent to IDH and 1p19q status, which can provide more precise management for IDH-mutant or 1p19q codeletion LGGs.

LLPS has emerged as a novel concept to elaborate the organization of living cells [6]. The potential prognostic value of LLPS-related molecular has also received increasing attention. Qiu et al. constructed a LLPS-related GS as a prognostic tool for epithelial ovarian cancer [17]. Our analysis might firstly develop the novel LLPS-related GS for predicting the prognosis of LGG. The LLPS-related GS was constructed using nine genes (ABL1, AR, CDK1, DAXX, ELN, KMT2D, POU5F1, SH3KBP1, and SYN1). Actually, functional studies of these nine genes in LGG are few. This suggests that it is helpful for our discovery of candidate key molecules of LGG from the perspective of LLPS. The associations of these genes to other cancers may help us study their functions in LGG. It was reported that ABL1 promotes cancer cell growth, survival, adhesion, and migration depending on the cellular context [32]. AR has been found to be associated with the occurrence, progression, prognosis, and drug resistance of ovarian cancer, endometrial cancer, and cervical cancer [33]. The upregulation of CDK1 can promote the growth and the proliferation of melanoma tumor cells [34]. SH3KBP1 was considered promoting glioblastoma tumorigenesis by activating EGFR signaling [35]. Deregulated expression of SYN1 may maintain a cancer stem-like phenotype that contributes to the development of gliomas [36]. POU5F1 was reported play a carcinogenic role in liver hepatocellular carcinoma [37]. Whether these genes affect the prognosis of LGG through a similar mechanism or just through the LLPS mechanism remains to be further investigated.

According to the results, the high-risk groups may have more active angiogenesis and may benefit from antiangiogenic treatment, such as bevacizumab [38]. We also found that the inflammatory response was more active in the high-risk LGG. Whether immunotherapy is more effective in these subgroups deserves further study [39]. We also found that other cancer-related pathways were enriched in the high-risk group. The more active epithelial-mesenchymal transition in the high-risk group may contribute to its biological behavior more similar to other cancers. Not surprisingly, we found case reports of patients benefiting from other unconventional treatments. Our analysis may provide a reference for the identification of these patients.

Although our present study may provide a new insight into the LLPS and LGG and the LLPS-related GS may improve the management of LGG, there were several notable limitations. Firstly, the LLPS-related GS was proposed based a retrospective study; it requires prospective studies to validate or even improve before going into clinical decision-making. Secondly, our study is mainly focused on Chinese LGG; whether the results are applicable to other races needs further study. Thirdly, molecular function experiments were lacking in the present study; thus, it remains elusive whether these LLPS-related genes are causal or merely prognostic markers in LGG.

## 5. Conclusion

In conclusion, we developed and validated a novel LLPS-related GS for risk stratification of LGG. Our findings may provide more precise management for LGGs and a useful reference for LLPS mechanism to link LGG studies.

## Figures and Tables

**Table 1:** The prognosis-related phase separation-related genes.

Predictor	Univariable Cox regression analysis				LASSO Cox coefficient	Multivariable stepwise Cox regression analysis			
	*β*	HR	95% CI for HR	*P* value		*β*	HR	95% CI for HR	*P* value
ABL1	0.212	1.237	1.027-1.49	0.025	-0.001241	-0.376	0.686	0.457-1.031	0.07
AKAP8	0.451	1.571	1.16-2.126	0.003	0				
AR	0.322	1.38	1.213-1.57	0	0.11761	0.171	1.187	1.006-1.399	0.042
CCNT1	0.219	1.245	1.038-1.494	0.018	0				
CDK1	0.24	1.271	1.133-1.427	0	0.148498	0.319	1.376	1.179-1.607	0
CPLX1	-0.227	0.797	0.729-0.871	0	-0.035502				
DAXX	0.323	1.381	1.02-1.871	0.037	-0.201314	-0.567	0.567	0.307-1.05	0.071
DNAJB1	0.232	1.261	1.009-1.574	0.041	0				
ELN	0.227	1.254	1.081-1.455	0.003	0.077678	0.154	1.167	0.963-1.414	0.115
EN2	0.156	1.169	1.048-1.303	0.005	0.044444				
G3BP1	0.236	1.266	1.068-1.5	0.006	0				
GOLGA2	0.422	1.525	1.174-1.981	0.002	0				
HNRNPA2B	0.259	0.259	1.043-1.61	0.019	0				
HNRNPAB	0.35	1.419	1.117-1.804	0.004	0				
HNRNPF	0.347	0.347	1.152-1.739	0.001	0				
HNRNPH1	0.317	1.373	1.146-1.646	0.001	0				
HTR1A	-0.122	0.885	0.828-0.946	0	-0.042494				
KMT2D	0.293	1.341	1.122-1.602	0.001	0.120985	0.344	1.41	1.082-1.837	0.011
KPNA2	0.205	1.228	1.02-1.478	0.03	0				
KPNB1	0.196	1.217	1.023-1.447	0.027	0				
MLLT1	0.163	1.177	1.029-1.348	0.018	0				
NUP98	0.283	1.327	1.049-1.68	0.018	0				
PMEL	0.214	1.239	1.072-1.431	0.004	0				
POU5F1	0.134	1.143	1.015-1.288	0.028	0.067597	0.117	1.124	0.976-1.294	0.105
PRNP	-0.155	0.856	0.75-0.978	0.022	0				
PTBP1	0.594	1.811	1.344-2.44	0	0				
RAD23B	0.234	1.264	1.012-1.578	0.039	0				
SH3KBP1	0.416	1.516	1.18-1.946	0.001	0.098287	0.331	1.392	0.983-1.972	0.063
SNCB	-0.178	0.837	0.778-0.9	0	0				
SOX2	0.169	1.184	1.016-1.379	0.03	0				
SPOP	0.209	1.232	1.014-1.497	0.036	0				
SYN1	-0.279	0.756	0.686-0.834	0	-0.13603	-0.274	0.76	0.676-0.854	0
SYN2	-0.188	0.828	0.754-0.91	0	0				
TNPO1	0.194	1.214	1.027-1.436	0.023	0				
USH1C	-0.154	0.857	0.796-0.922	0	-0.07669				
WWTR1	0.232	1.261	1.102-1.443	0.001	0				
YAP1	0.246	1.279	1.114-1.468	0	0				

Abbreviations: HR: hazard ratio; CI: confidence interval; LASSO: least absolute shrinkage and selection operator.

## Data Availability

The raw analyses from this study can be obtained from the corresponding author upon reasonable request.
